# Gene expression and cellular changes in injured myocardium of *Ciona intestinalis*

**DOI:** 10.3389/fcell.2024.1304755

**Published:** 2024-03-13

**Authors:** Serenity Stokes, Pooja Pardhanani Palmer, Jeremy L. Barth, Robert L. Price, Bella G. Parker, Heather J. Evans Anderson

**Affiliations:** ^1^ Central Piedmont Community College, Natural Sciences Division, Charlotte, NC, United States; ^2^ Atrium Health, Division of Community and Social Impact, Department of Community Health, Charlotte, NC, United States; ^3^ Department of Regenerative Medicine and Cell Biology, Medical University of South Carolina Proteogenomics Facility, Charleston, SC, United States; ^4^ Department of Cell Biology and Anatomy, University of South Carolina School of Medicine, Columbia, SC, United States; ^5^ Department of Biology, Stetson University, DeLand, FL, United States; ^6^ Department of Health and Biomedical Sciences, Advent Health University, Orlando, FL, United States

**Keywords:** *Ciona intestinalis*, myocardium, gene expression, cardiac myocyte, microarray

## Abstract

*Ciona intestinalis* is an invertebrate animal model system that is well characterized and has many advantages for the study of cardiovascular biology. The regulatory mechanisms of cardiac myocyte proliferation in *Ciona* are intriguing since regeneration of functional tissue has been demonstrated in other organs of *Ciona* in response to injury*.* To identify genes that are differentially expressed in response to *Ciona* cardiac injury, microarray analysis was conducted on RNA from adult *Ciona* hearts with normal or damaged myocardium. After a 24- or 48-h recovery period, total RNA was isolated from damaged and control hearts. Initial results indicate significant changes in gene expression in hearts damaged by ligation in comparison to control hearts. Ligation injury shows differential expression of 223 genes as compared to control with limited false discovery (5.8%). Among these 223 genes, 117 have known human orthologs of which 68 were upregulated and 49 were downregulated. Notably, *Fgf9/16/20*, insulin-like growth factor binding protein and Ras-related protein *Rab11b* were significantly upregulated in injured hearts, whereas expression of a junctophilin ortholog was decreased. Histological analyses of injured myocardium were conducted in parallel to the microarray study which revealed thickened myocardium in injured hearts. Taken together, these studies will connect differences in gene expression to cellular changes in the myocardium of *Ciona*, which will help to promote further investigations into the regulatory mechanisms of cardiac myocyte proliferation across chordates.

## Introduction

As a tunicate, *Ciona intestinalis* is part of the group of invertebrate chordates that are most closely related to vertebrates (Delsuc et al., 2006). Molecular phylogenetic analyses indicate that much of the *Ciona* protein-encoding genome is conserved when compared to vertebrates, but with reduced genetic redundancy (Dehal et al., 2002). Current literature and database resources such as Aniseed (https://www.aniseed.cnrs.fr/) provide extensive information on the *Ciona* genome annotations, gene expression patterns and developmental lineages of major cell types, including central and peripheral nervous system and the cardiopharyngeal lineage (Stolfi et al., 2010; Abitua et al., 2012; Stolfi et al., 2015; Brozovic et al., 2018; Horie et al., 2018). Moreover, *Ciona* embryos develop rapidly and are easily manipulated with molecular tools (Davidson, B. 2007; Cota et al., 2017). Thus, *Ciona* as part of the closest invertebrate outgroup to the vertebrates is an excellent animal model for the analysis of gene function and developmental processes.

While most studies investigating *Ciona* have examined embryonic and larval phases, few studies have examined mature adults. The adult *Ciona* is a sessile marine organism whose average size varies from 12 to 20 cm in length with the major organs located in the caudal region and the heart lying adjacent to the stomach. While the cardiac cells are specified during embryonic development, the heart forms post-metamorphosis during the juvenile phase (Davidson, 2007; Cota et al., 2017). Cardiac myocytes in *Ciona* share many of the same structural features as other chordates; however, the myocardium of *Ciona* is comprised of a single layer of cardiac myocytes *versus* the trabeculated myocardium of more complex chordates (Evans Anderson and Christiaen, 2016). The ultrastructure of the adult *Ciona* myocardium was described previously (Evans Anderson and Christiaen, 2016). This review suggests that the *Ciona* heart retains the genetic underpinnings of cardiac cell specification and basic cellular organization for all chordates. Of note, cardiac myocytes in *Ciona* are mononuclear and have less sarcomeric organization than mature mammalian cardiac myocytes. Instead, the ultrastructure of *Ciona* cardiac myocytes is more similar to embryonic or neonatal cardiac myocytes in mammals (Goldstein and Traeger, 1984; Evans Anderson and Christiaen, 2016; Guo and Pu, 2020). This is interesting since mononuclear cardiomyocytes in mammals have been shown to retain proliferative abilities, whereas mature polyploid cardiac myocytes lose proliferative capacity (Shen et al., 2020).

Regeneration of functional tissue has been characterized in *Ciona*. Studies have shown complete regeneration of siphons and neural complex (Dahlberg et al., 2009; Jeffery, 2015). However, regeneration of the *Ciona* heart has not been demonstrated yet, but it remains likely given that other solitary ascidians are capable of complete regeneration of tissues, including the heart (Gordon et al., 2021). In this study we examine altered gene expression and cellular changes to injured myocardium of *Ciona*. Very few studies have been conducted on the *C. intestinalis* heart and no previous studies have detailed the effects of cellular injury to the *C. intestinalis* heart. As the most closely related group to vertebrates with orthologous genes and conserved cell lineage specification mechanisms, *Ciona* can provide important insights into cardiac gene regulation, as well as cardiac myocyte biology.

## Materials and methods

### Animal husbandry

*Ciona intestinalis* adults (7–12 cm) were obtained by M-REP (San Diego, CA). The animals were maintained in a 65-gallon saltwater tank equipped with appropriate water circulation and filtration system.

### Injury model

Adult *Ciona* hearts were subjected to injury via ligation. To set up ligations, hearts were exposed via a 3 cm incision in the animal’s tunic and body wall muscle. Surgical silk (4 cm pieces of 000 gauge (Ethicon, NJ)) was tied around the exposed heart apex and tightened to restrict fluid flow but allow peristaltic contractions to continue. Animals were placed in separate baskets for a 24- or 48-h recovery period. At the end of each of these periods, hearts that were still visibly beating were processed for analysis. Control groups consisted of exposed but non-ligated hearts for each treatment group (24- and 48-h). A minimum of 4 hearts per group was done for each experiment, which were repeated at least three times for each group.

### Histology

Ligated and control hearts were fixed in 4% paraformaldehyde (Electron Microscopy Sciences, PA) and stored at 4°C. Heart tissues were placed in a tissue processor, dehydrated in a graded ethanol series and embedded in Paraplast Xtra (Electron Microscopy Sciences, PA). Sections were cut at 5 mm and stained with Harris hematoxylin, immunofluorescence or Modified Russell-Movat Pentachrome staining. For immunostaining, slides were rehydrated through a graded series of MeOH/PBS. (75%, 50%, 25%) for 15 min each and then washed twice with PBS. PBSBT1 (1%–5% BSA, 1% Triton X-100 in PBS) was used to block for 1 h at RT twice. Diluted primary antibody (1:100 MF20) in PBST2 (1% BSA, 0.5% Triton X-100 in PBS) was applied and slides were incubated at 4°C for 16 h and then washed in PBST for 30 min five times. Secondary antibody with fluor was diluted in PBST2 and incubated for 4 h at RT then washed in PBST for 30 min five times. DAPI was included in mounting media. Slides were imaged using confocal microscopy.

### Modified Russell-Movat Pentachrome staining

Modified Russell-Movat Pentachrome Stain (ScyTek Laboratories, UT) was used to histologically visualize the presence of muscle, mucin, collagen, elastin, and fibrin in *Ciona* heart sections. Staining incubations were performed at room temperature. A working elastic stain solution (ESS) was prepared using 2 parts hematoxylin solution (5%), 1-part ferric chloride solution (10%), and 1 part Lugol’s Iodine solution. For 10 slides, 20 drops of hematoxylin, 10 drops of ferric chloride, and 10 drops of Lugol’s Iodine were, and sections stained for 20 min. Slides were rinsed in running tap water until no excess stain remained. The slides were laid in the staining tray at an angle, and 15 drops of ferric chloride (2%) differentiating solution were applied and allowed to run off the slides. The slides were rinsed in running tap water, and then checked with a light microscope for differentiation of the tissue. If differentiation was not seen, the ferric chloride (2%) differentiation solution was applied again and allowed to run off the slides. The slides were then rinsed in two changes of distilled water. Next, 10 drops of sodium thiosulfate solution (5%) were applied to slides and incubated for 1 min. The slides were then rinsed in tap water for 2 min followed by 2 successive 1-min rinses in distilled water. 10 drops of acetic acid solution (3%) were applied, and the slides were incubated for 2 min to equilibrate the tissue for alcian blue staining. The acetic acid solution was shaken off, and without rinsing, 10 drops of alcian blue solution, pH 2.5, were applied. The slides were incubated for 25 min, rinsed in tap water for 2 min followed by 2 successive 1-min changes in distilled water. 10 drops of Biebrich Scarlet/Acid Fuschin solution were applied. The slides were incubated for 10 min, and then rinsed in two 1-min changes of distilled water. Next, the tissue was equilibrated with a 10 s application of acetic acid (1%) with agitation and rinsed quickly in distilled water. Slides were then differentiated in 2 changes of 10 drops of phosphotungstic acid (5%) for 3 min each. The slides were then analyzed microscopically for proper differentiation of elastic fibers (collagen fibers remained clear at this checkpoint); if this was not achieved, the differentiation process was repeated. The slides were then placed back in a tray at an angle, and 10 drops of acetic acid (1%) were applied and allowed to run over the sections and off the slides. The slides were laid flat again, and without rinsing, 10 drops of tartrazine were applied. The slides were incubated for 1 minute and then rinsed in three 1-min changes of absolute alcohol.

### Microarray

RNA was isolated by RNeasy Mini Kit (Qiagen) from injured and control hearts at 24 h or 48 h following treatment. Biological replicates were prepared for each sample type (control 24 h *n* = 3; control 48 h *n* = 2; injured 24 h *n* = 4; injured 48 h *n* = 3). A custom *Ciona* microarray A-AFFY-106 (CINT06a520380F; Affymetrix, Affymetrix, Santa Clara, CA) was obtained in collaboration with L. Christiaen (New York University). RNA samples were processed for Affymetrix microarray analysis at the Medical University of South Carolina Proteogenomics Facility as previously described (Lang et al., 2015). Briefly, total RNA samples were evaluated by Bioanalyzer 2100 (Agilent) to ensure integrity of ribosomal peaks and absence of degradation products. RNA was converted to biotin-labeled, fragmented cRNA using the 3′ IVT Express Kit (Affymetrix) according to manufacturer recommendations. Labeled cRNA was hybridized to GeneChips overnight at 45°C in a rotating oven. Post hybridization washing, staining and fluorescence scanning were performed with Affymetrix instrumentation and reagents as directed. Hybridization data (CEL files) were imported into Affymetrix Expression Console software and intensity values normalized by robust multi-array average (RMA) (Irizarry et al., 2003). Comparative analysis was done with dChip software (Li and Wong, 2001). Criteria for differential expression were absolute fold change greater >2 and *p* < 0.01 (Student’s t-test). False discovery was estimated based on the number of genes discovered by iterative comparison with permuted sample assignments. Annotations for differentially expressed genes were accessed via the Aniseed Database. Raw microarray data (CEL files) are deposited at NCBI Gene Expression Omnibus (Accession # GSE244713).

### Reverse transcription quantitative PCR (RT-qPCR)

RNA isolated from embryos, larvae, and juvenile *Ciona* as well as control (*n* = 4) and injured adult *Ciona* hearts (*n* = 6) was converted to cDNA using iScript™ cDNA Synthesis Kit (Bio-Rad) following manufacturer’s protocol, then stored at −80°C until qPCR analysis. Primers were selected using Primer3 program (http://frodo.wi.mit.edu/) for *Ciona* Fgf9/16/20 (GenBank: AB086097.1) and *Ciona* FoxP genes (GenBank: NM_001078471.1) (Table 1). *Ciona* β-actin (CLSTR00046) expression was used as an internal control to normalize expression data. Serial dilutions of cDNA were used to evaluate primer efficiency. *Ciona* cDNA was diluted 1:10 for use in qPCR reaction. The reaction mix was 2 µL diluted cDNA; 5 μL L 2x SyBr Green PCR mix (iTaq universal SYBR green supermix, Bio-Rad); 0.05 µL 10 mM forward primer; 0.05 µL 10 mM reverse primer; and 2.9 µL nuclease free water. Reactions were run using Bio-rad CFX384 qPCR machine with the following protocol: 95°C 30 s; then 40 cycles of 95°C 5 s; 60°C 1 min. Amplifications were performed in triplicate. The average Ct value for β-actin was used to normalize the Ct values for FGF and FoxP genes. Each assay included a no-template control and a reverse transcription negative control for each primer pair. Data were analyzed using Bio-Rad CFX Maestro Software.

**TABLE 1:** Primer sets.

Primer	Sequence
FGF 131	Left: TCA​ATA​CGC​CGT​CCT​TGA​AT
	Right: ACT​CGC​GAT​TGA​ATG​TTT​C
FGF 113	Left: GGT​ACC​CAA​GAA​AGC​CAC​AA
	Right: TTG​CTG​TTC​ATT​GCC​AGG​TA
FoxP 148	Left: TAG​CCC​AGA​AGA​GGA​CGG​TA
	Right: CTG​TTG​CCG​GTA​AAA​TCC​GT
FoxP 165	Left: CAT​GGA​AGA​ACG​CTG​TGA​GA
	Right: ACG​GAG​TTT​CGA​TAC​GGT​TG

## Results

**FIGURE 1:**
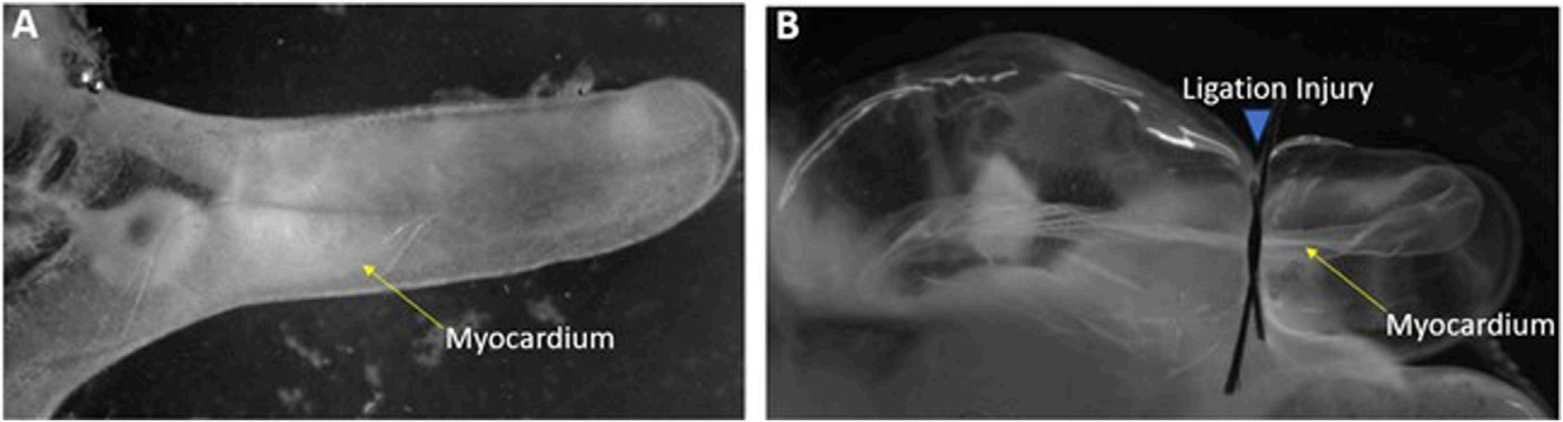
Ligation injury model of Ciona heart **(A)** Control heart exposed from *Ciona*. **(B)** Injured heart following 24-h of ligation.

To elucidate genes that might be involved in induction of cardiac myocyte proliferation and myocardial regeneration, a microarray was conducted on control *versus* injured *Ciona* heart in to examine gene expression changes. To induce cardiac injury, adult *Ciona* hearts were exposed from the tunic. Injury to the hearts was done by ligation in which a surgical thread was tied around the heart to restrict flow for periods of 24- or 48-h (Figure 1). The contraction of the myocardium was used to confirm the animal was still alive post-injury. Several methods of injury to the *Ciona* hearts were attempted and ligation was the injury that allowed the animals to live for 48–72 h post injury and also produced results that were evident histologically and replicable. Ligation has been used as a form of myocardial injury in other animal models (Abarbanell et al., 2010). Injured and control hearts were then dissected and used to isolate RNA or fixed for further histological analyses.

**FIGURE 2:**
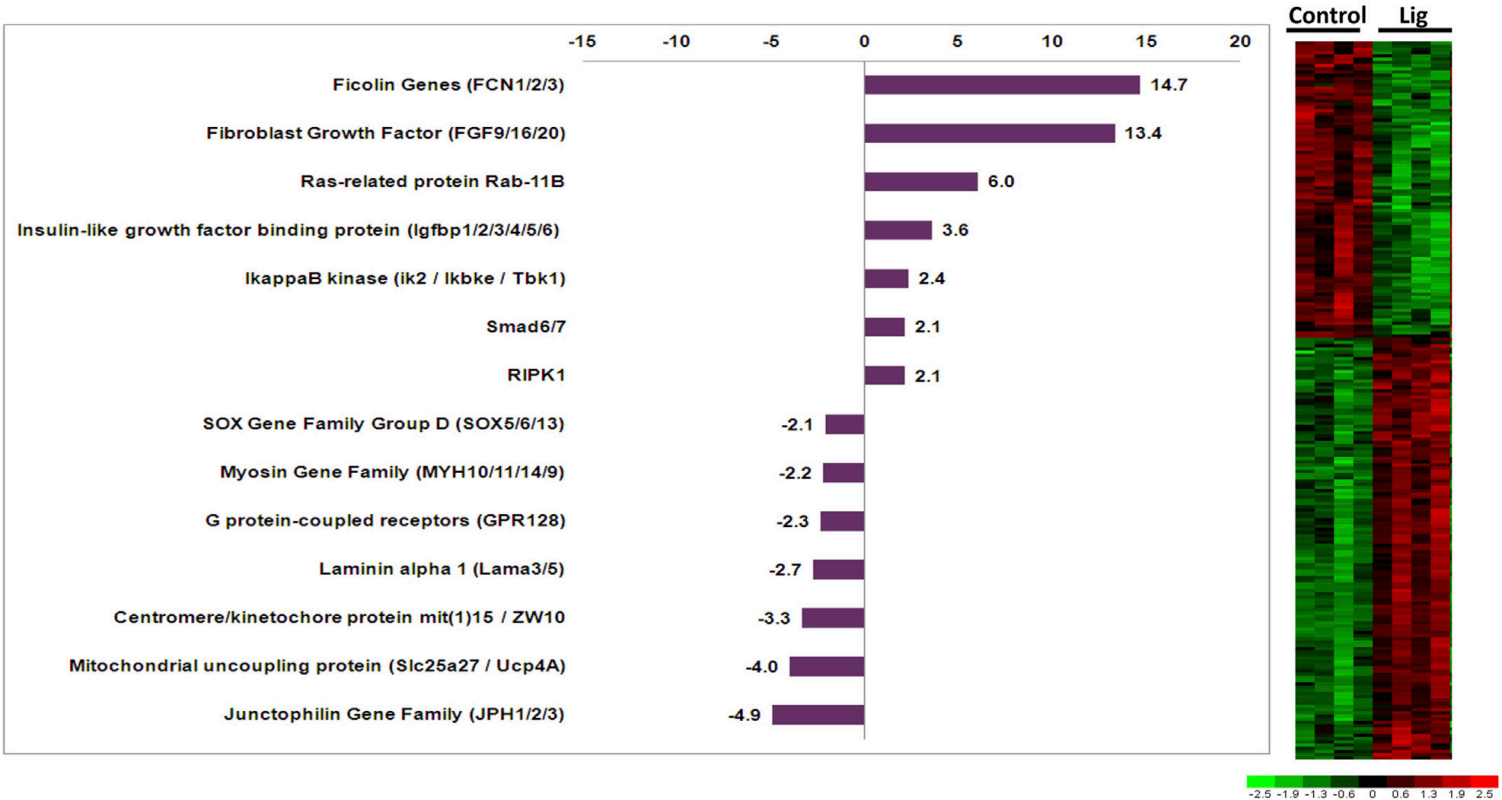
Altered gene expression in injured *Ciona* hearts. Comparison of ligated *versus* control hearts identified 223 differentially expressed genes. Fold changes for select orthologous genes are shown in the graph. Expression profiles for all differentially expressed genes are illustrated in the adjacent heatmap.

RNA isolated from control *versus* injured hearts was subjected to microarray analysis via custom Affymetrix GeneChips. Comparisons restricted to 24 or 48-h time points did not yield significant genes. Therefore the 24- and 48-h samples within each group were treated as the same sample type. Results of differential expression analysis are illustrated in the adjacent heatmap (Figure 2). Analysis of ligated hearts *versus* control yielded 223 differentially expressed genes (fold change >2, p < 0.01, Student’s t-test) with limited false discovery (5.8%). Among these 223 genes, 117 have known human orthologs of which 68 were upregulated and 49 were downregulated. The annotations for the differentially expressed genes were accessed via the Aniseed Database (http://www.aniseed.cnrs.fr/). Expression changes for genes of interest are shown in the graph below (Figure 2). *Fgf9/16/20* is of particular interest, along with other proliferation related genes such as *Rab11b* and insulin like growth factor binding protein.

The full data set is accessible via Gene Expression Omnibus (GEO) – NCBI, to review GEO data, use accession GSE244713: Go to: https://nam10.safelinks.protection.outlook.com/?url=%3A%2F%2Furldefense.com%2Fv3%2F__%3A%2F%2Fwww.ncbi.nlm.nih.gov%2Fgeo%2Fquery%2Facc.cgi%3Facc%3DGSE244713__%3B!!Ab1_Rw!Fm1KQOqKhUyHpx-ej14R43nOCMBeCjIXhLQRxlrqMdwvV1j4UOc4s7_tEVpsxcIpIhjLdFqeIoQ_98XWpg%24&data=05%7C01%7Chevansanderson%40stetson.edu%7C99b4895e5b9d443e0c8408dbc9ad6557%7C7d854659421348c180cadf7831c02e6d%7C0%7C0%7C638325518791474686%7CUnknown%7CTWFpbGZsb3d8eyJWIjoiMC4wLjAwMDAiLCJQIjoiV2luMzIiLCJBTiI6Ik1haWwiLCJXVCI6Mn0%3D%7C3000%7C%7C%7C&sdata=XS1LE4cBd02SmDPJ7qfKRhU%2FPeehACqPJA9ZV9Tv2CY%3D&reserved=0. Enter token ipwhooiexjmhvgp into the box.

**FIGURE 3:**
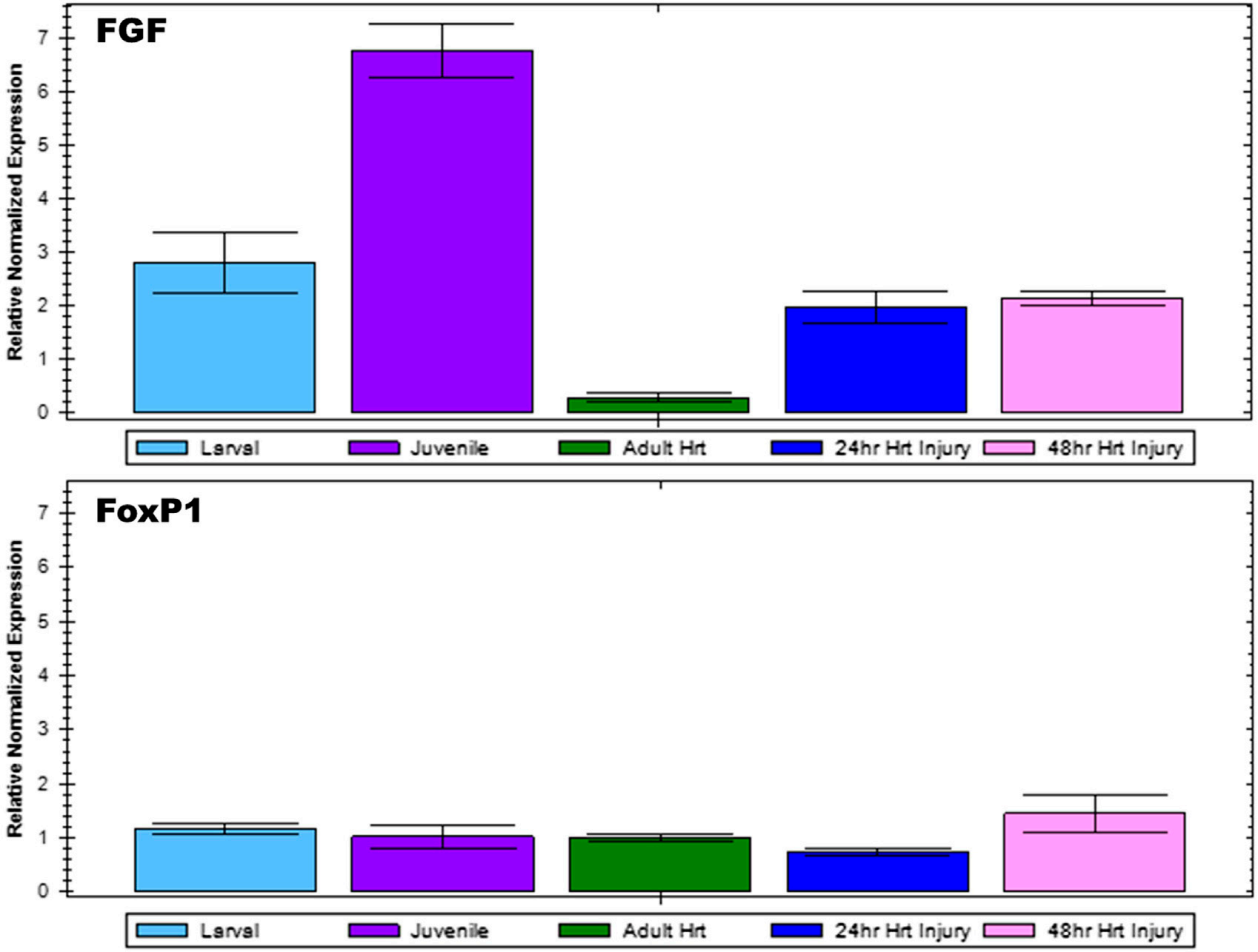
qPCR analysis to confirm microarray results. Relative normalized expression levels of *Fgf9/16/20* and *FoxP1* for *Ciona* larval, juvenile, and adult heart as well as injured adult heart at 24 and 48-h post-injury were analyzed by RT-qPCR. Results are normalized relative to *β-Actin*. Error bars represent standard error of the mean.

To confirm microarray analysis results, select genes of interest were subjected to reverse transcription quantitative PCR. *Fgf9/16/20* gene expression was analyzed for *Ciona* larval, juvenile, and adult hearts as well as injured adult hearts at 24 and 48 h post injury (Figure 3). Gene expression levels were normalized using β-Actin expression for each sample. *Fgf9/16/20* expression was found to be lowest in the adult heart; however, during the juvenile stage when the heart is forming, *Fgf9/16/20* is most highly expressed. When the heart is injured, *Fgf9/16/20* expression increased compared to adult heart samples in both 24- and 48-h ligation injury time points. In comparison, expression of *FoxP1* was examined across all samples. *FoxP1* did not significantly change across developmental timepoints or with injured hearts.

**FIGURE 4:**
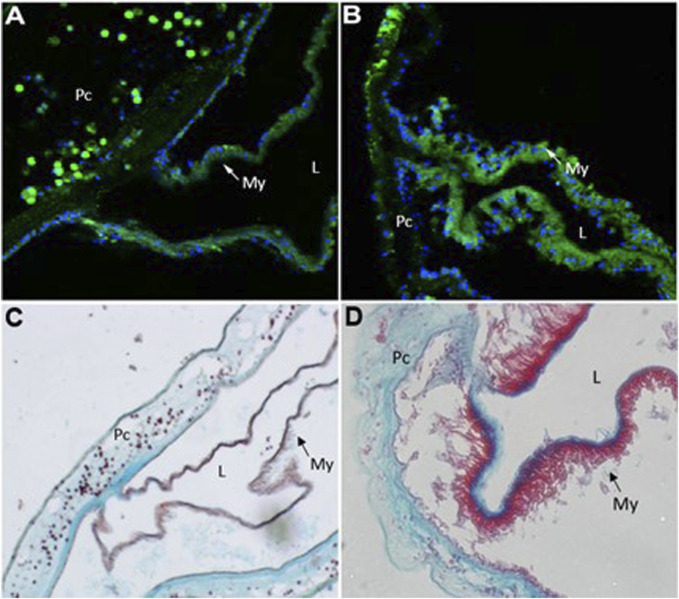
Injury to heart results in cellular changes to myocardium. Representative images of control **(A and C)** *versus* injured **(B and D)** *Ciona* hearts. **(A)** section of control heart showing the myocardium (My,green) where it transitions to the pericardium (Pc). Punctate green bodies were observed in the pericardial space. Cardiac myocyte nuclei (blue) are seen at the basal side of the myocardium opposite of the lumen (L). **(B)** injured myocardium (green) increases sarcomeric myosin expression after 48 h of injury. **(C)** control heart stained with Movat’s Pentachrome (Muscle–RED, Mucins–BLUE, Elastin–BLACK/BLUE, Collagen–YELLOW, Fibrin–intense Red) shows where the myocardium (My, red) joins the pericardium (Pc). Punctate red bodies were observed in the pericardial space **(D)** injured myocardium (red) appears thickened 48 h post injury.

In order to examine the cellular changes that occurred after cardiac injury in *Ciona*, histological analyses were conducted. Immunofluorescence was performed using MF20 antibody to detect myosin heavy chain in cardiac myocytes (Bader et al., 1982) and DAPI to visualize nuclei (Figure 4). After injury, the myocardium thickens as visualized by increased MF20 expression (Figure 4B). This result was consistent in sections stained with Movats Pentachrome stain, which identifies collagen, elastin, muscle, mucin and fibrin in tissue sections (Figure 4D). These histological assays also revealed punctate bodies in the pericardium of *Ciona* hearts that were labeled by both MF20 and as muscle in Movats Pentachrome staining. These bodies were most defined in the control sections (Figures 4A,C). Further analysis is required in order to understand what these punctate bodies are and how the structural changes observed in the myocardium compare to responses to cardiac injury in other species.

## Discussion

The *Ciona* model system presents an interesting opportunity to study cellular structure and gene expression changes in an adult myocardium. *Ciona* Comparison of the gene changes observed in *Ciona versus* gene expression changes that occur in experiments with more complex hearts might reveal interesting results. Moreover, *Ciona* may provide a chance to delineate the cellular and molecular requirements for cardiac regeneration with two distinct advantages: a chance to track cellular origins back to early progenitors and the genetic and cellular simplicity of an invertebrate chordate.

Here, we utilized a microarray study to analyze gene expression changes that occurred after injury to the adult heart. One of the most upregulated genes Fgf9/16/20, which increased 13.4 fold in injured hearts, has been shown in vertebrates to be an important regulator of cardiac myocyte proliferation (Itoh et al., 2016; Khosravi et al., 2021; Tahara et al., 2021). Interestingly, Fgf16/20 in mice regulates proliferation via cell signaling from the endocardium and cell signaling from the epicardium in other vertebrates (Lavine et al., 2005; Smith and Bader, 2007). However, *Ciona* do not have a discernable endocardium or epicardium but might retain some of the cell signaling mechanisms from Fgf 9/16/20 that promote cardiac myocyte proliferation. Furthermore, it has been shown that the FGF pathway is critical in the specification of progenitor cells to the cardiac lineage in *Ciona* as well as vertebrates (Davidson et al., 2006; Woznica et al., 2012; Razy-Krajka et al., 2018; Tahara et al., 2021). Fgf 9/16/20 has been reported to be part of the subfamily E, which shows clear evolutionary relationship that is shared among chordates (Oulion et al., 2012). Other notable genes included insulin-like growth factor binding protein (IGFbp) and Rab11b (3.6-fold and 6-fold upregulated, respectively) since these genes are also involved in cardiac development, regeneration, and induction of pluripotent stem cells to cardiac myocytes (Sugden, 2003; Díaz del Moral et al., 2021). IGFbp has also been implicated in the inhibition of apoptosis in cardiac myocytes (Tang et al., 2021). The IGFbp gene family has a complex evolution where current evidence suggests that these genes evolved distinct functions early in vertebrate evolution (Daza et al., 2011). Additional characterization of IGFbp ortholog functions across chordate species would help elucidate how specific functions arose. Rab proteins are known to regulate vesicular transport in mammalian cardiac myocytes which are important in cell signaling mechanisms related to cardiac hypertrophy (Wu et al., 2001; Elias et al., 2012). Specifically, Rab11 is involved with GLUT4 glucose transporter in response to insulin in vertebrates (Kessler et al., 2000). The *Rab* family is well conserved across all chordates and is thought to have a predominant role in molecular communication between cells (Coppola et al., 2019). Notable genes that were found to be downregulated include the junctophilin gene family ortholog, which decreased 4.9-fold in ligated hearts. Decreased junctophilin expression in mice and humans has been found to cause cardiac hypertrophy (Landstrom et al., 2011; Beavers et al., 2014). Thus, the observed gene expression changes in injured *Ciona* hearts include orthologous genes in vertebrates that are known to have important roles in cardiac development and disease processes.

As part of the closest invertebrate outgroup to the vertebrates with a conserved genetic, the Ciona heart provides a useful model system to further elucidate the blueprint of cardiac myocyte proliferation and potentially provide insights into conserved cardiac regeneration mechanisms. Genetic manipulation or alteration of cell signaling mechanisms could be used in Ciona to promote cardiac myocyte proliferation or myocardial growth using conserved signaling pathway components and/or growth factors. The easily accessible Ciona heart could be used to study effects of flow and mechanotransduction on cardiac myocytes. The possibilities using modern techniques to further characterize the Ciona heart are vast and these studies may provide important insights into cardiac myocyte biology.

## Data Availability

The original contributions presented in the study are publicly available. This data can be found here: GSE244713.
